# Credit and blame for AI–generated content: Effects of personalization in four countries

**DOI:** 10.1111/nyas.15258

**Published:** 2024-11-25

**Authors:** Brian D. Earp, Sebastian Porsdam Mann, Peng Liu, Ivar Hannikainen, Maryam Ali Khan, Yueying Chu, Julian Savulescu

**Affiliations:** ^1^ Uehiro Oxford Institute University of Oxford Oxford UK; ^2^ Centre for Biomedical Ethics, Yong Loo Lin School of Medicine National University of Singapore Singapore; ^3^ Centre for Advanced Studies in Bioscience Innovation Law Faculty of Law University of Copenhagen Copenhagen Denmark; ^4^ Faculty of Law University of Oxford Oxford UK; ^5^ Center for Psychological Sciences Zhejiang University Hangzhou China; ^6^ Department of Philosophy I, Faculty of Psychology University of Granada Granada Spain; ^7^ Department of Psychology and Behavioral Sciences Zhejiang University Hangzhou China

**Keywords:** credit–blame asymmetry, generative artificial intelligence, large language model, moral responsibility, personalization

## Abstract

Generative artificial intelligence (AI) raises ethical questions concerning moral and legal responsibility—specifically, the attributions of credit and blame for AI‐generated content. For example, if a human invests minimal skill or effort to produce a beneficial output with an AI tool, can the human still take credit? How does the answer change if the AI has been personalized (i.e., fine‐tuned) on previous outputs produced without AI assistance by the same human? We conducted a preregistered experiment with representative sampling (*N* = 1802) repeated in four countries (United States, United Kingdom, China, and Singapore). We investigated laypeople's attributions of credit and blame to human users for producing beneficial or harmful outputs with a standard large language model (LLM), a personalized LLM, or no AI assistance (control condition). Participants generally attributed more credit to human users of personalized versus standard LLMs for beneficial outputs, whereas LLM type did not significantly affect blame attributions for harmful outputs, with a partial exception among Chinese participants. In addition, UK participants attributed more blame for using any type of LLM versus no LLM. Practical, ethical, and policy implications of these findings are discussed.

## Graphical abstract

This study examined how personalized large language models (LLMs) affect laypeople's attributions of credit and blame for AI‐generated content. In a preregistered experiment (*N* = 1802) across four countries, personalized LLMs increased credit for beneficial output but did not impact blame for harmful writing, with some national differences. Personalization could impact human–AI collaboration by influencing perceived moral responsibility of human users for cocreated outputs.

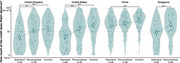

## INTRODUCTION

Since the introduction of large language models (LLMs), generative artificial intelligence (AI) has become a focal point of debate.^1^ The impressive generative capabilities of LLMs enable the production of high‐quality outputs.^2^ However, this technology is not without its challenges: It has the potential to generate both beneficial and harmful content.^3^ Whether positive or negative, the content generated by AI results from the interaction between the prompting human and the AI model.^4^ Consequently, ethical questions arise, particularly as to AI users’ moral responsibility, including how much credit or blame they deserve for AI‐generated content.^5^, ^6^, ^7^ Previous work in moral psychology has explored the relationship between praise and blame judgments primarily in response to technologically unassisted human behavior.^8^, ^9^ The impact of human use of generative AI on this relationship is not well understood.^2^, ^3^

### Credit–blame asymmetry and personalization

When technologies enable achievements with reduced human effort, moral credit assigned to human users may likewise be reduced.^10^ Drawing on previous work, we argue it should be harder to earn full credit for jointly producing a positive outcome with generative AI insofar as relatively less effort, skill, or creativity is required on the part of the human user.^11^ Yet the threshold for blame, we have suggested, may be unaffected insofar as recklessness or negligence in bringing about harm can be sufficient for high levels of blame regardless of AI use.^5^ Hence, we have hypothesized that the use of generative AI to produce a given output “elevates the bar for earning credit, but standards for assigning blame remain the same”: that is, use of generative AI entails a credit–blame asymmetry.^5^

This asymmetry has significant implications. On the one hand, it foreshadows the emergence of achievement gaps^10^ for humans: Valuable or beneficial outputs will be produced due to human use of generative AI, but many of these outputs will not be creditable as human achievements. On the other hand, when AI use leads to harmful outcomes, the blame attributed to humans may not diminish. This could disincentivize the use, or acknowledgment of use, of generative AI by humans.

Here, we investigate the effects of *personalization* on credit and blame judgments for beneficial and harmful content generated using AI. We use personalization to refer to the process by which an AI, such as an LLM, is fine‐tuned on individual‐level data: for example, on one's own past writing or other creative work, as is becoming increasingly feasible for many users.^12^, ^13^ Because a personalized LLM system would be based on a user's *previou*s effort, skill, and so on—in contrast to a standard, off‐the‐shelf LLM such as OpenAI's ChatGPT—we predict that people will attribute more credit to human users for positive outcomes resulting from the use of such systems.

But then, personalized AI use might also lead to harmful outcomes.^13^ Will this result in greater blame to the human user? Although possible in some cases, when the harm is due to human negligence or recklessness in their use of AI, as we investigate here, the specific type of AI—personalized versus standard—might not make much of a difference. This is because the human might be seen as blameworthy for their carelessness in using AI, irrespective of personalization.

### Present research

The impact of personalization on credit and blame judgments for AI‐generated content is unknown. Building on previous work in moral psychology assessing lay attributions of credit and blame in relation to technologically unassisted human behavior,^8^ here we examine similar attributions in relation to AI‐assisted behavior, with an emphasis on the effects of personalization. We preregistered two hypotheses (https://aspredicted.org/B7J_KSX):

**H1**. For equivalent beneficial outcomes, more credit will be attributed to a human user when using a personalized versus standard LLM.
**H2**. For equivalent harmful outcomes, blame attributions will be comparable regardless of LLM type.

## METHODS

### Procedure and measures

**FIGURE 1:**
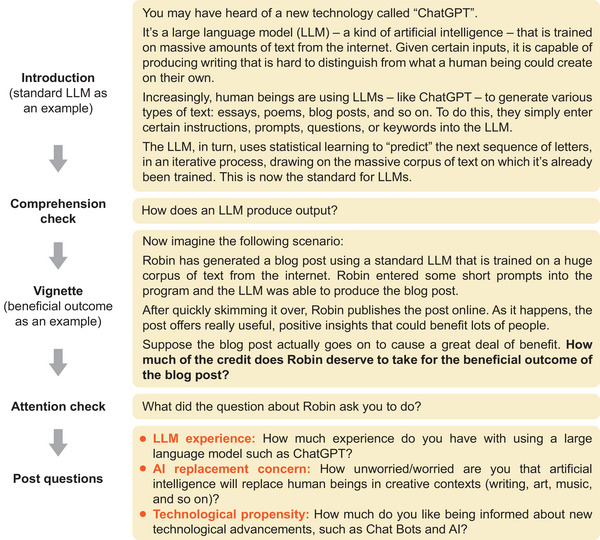
Experimental procedure in our surveys (using the standard large language model [LLM] condition with a beneficial outcome as an example).

We created six vignettes based on a 3 (content production method: personalized LLM vs. standard LLM vs. control condition in which no LLM is used) × 2 (outcome type: beneficial vs. harmful) between‐subjects design. In our LLM conditions, Robin, a fictitious character, was described as using a personalized LLM (fine‐tuned on Robin's previous writings) or a standard LLM such as ChatGPT to write a blogpost. In the control condition, Robin was described as writing the post manually using information from the internet (i.e., no LLM use). In all conditions, after quickly skimming over the blogpost, Robin publishes it online. In the beneficial outcome conditions, the post is filled with useful information that could be helpful to many people, whereas in the harmful outcome conditions, the post is filled with disinformation that could be harmful to many people. After reading the vignette, participants rated how much credit or blame (depending on the outcome type) Robin deserved on a scale from 0 (none at all) to 100 (all of it). Participants were also invited to explain their ratings in an open‐ended response box, though these qualitative data were not subjected to a formal analysis. Subsequently, they responded to three post‐experiment questions regarding their LLM experience, AI replacement concern, and technological propensity (see Figure 1) and reported their gender and age. An illustration of the study procedure is shown in Figure 1 (refer to the Supporting Information for the specific wording in each condition).

### Participants

Our preregistered experiment was repeated in two Western cultural zones (United Kingdom and United States) and two Eastern cultural zones (China and Singapore). We aimed to recruit about 600 participants per nation, as preregistered. Survey data were collected on Prolific in the United Kingdom and United States, Credamo in China, and REDcap in Singapore. Table S1 provides information on our final sample (*N* = 1802), which was nationally representative in terms of age and gender. Data collection occurred between July and September 2023.

From the initial 640 UK participants, we excluded data from 192 participants who failed an attention check (*n* = 9) or comprehension check (*n* = 150) or provided incomplete responses (*n* = 33), leaving 448 (*M*_age_ = 46.5, *SD*_age_ = 15.4; 220 women, 225 men, two identifying as “Non‐binary/Other” or “Prefer not to say”, and one who did not respond to the gender question).

Of the initial 644 US participants, we excluded data from 181 participants who failed an attention check (*n* = 22) or comprehension check (*n* = 119) or provided incomplete responses (*n* = 40), leaving 463 (*M*_age_ = 46.7, *SD*_age_ = 16.1; 236 women, 216 men, 10 identifying as “Non‐binary/Other,” and one who did not respond to the gender question).

Among the initial 667 Chinese participants, we excluded data from 63 participants who failed an attention check (*n* = 25) or comprehension check (*n* = 38), and 1 for not providing a numeric response to the age question, leaving 603 (*M*_age_ = 44.0, *SD*_age_ = 13.9; 304 women, 229 men).

Among the initial 682 Singaporean participants, we retained data from 466 participants in the 4 LLM conditions. Data from the two control conditions in the Singapore experiment were excluded prior to data analysis due to a programming error that caused participants to be shown information about LLMs (despite being in the no‐LLM condition) before submitting their credit/blame ratings (see Supporting Information notes for details). We also excluded data from 145 participants who failed an attention (*n* = 38) or comprehension (*n* = 107) check and from 33 participants who chose “I do not plan to read carefully, so you won't be able to use my data,” finally resulting in 288 (*M*_age_ = 46.5, *SD*_age_ = 13.4; 166 women, 122 men).

## RESULTS

As preregistered, we conducted a 3 (LLM type plus control) × 2 (beneficial or harmful outcome) analysis of variance (ANOVA) on the data from each collected sample, finding that in all conditions and surveyed countries, more blame was assigned to Robin for the harmful outcome than credit was assigned to Robin for the beneficial outcome, consistent with the theorized credit–blame asymmetry (see Supporting Information notes for details and caveats). However, given that the credit and blame ratings were collected on different scales, which may render such direct comparisons questionable, we conducted separate ANOVAs for each outcome, employing the Bonferroni method for pairwise comparisons. Moreover, given that participants’ individual difference scores (i.e., LLM experience, AI replacement concern, and technological propensity) might influence their credit and blame ratings, we also conducted analyses of covariance (ANCOVA), treating these individual differences as covariates. This allowed us to determine the robustness of our results. In our preregistration, we did not consider participants’ origin (i.e., country) as an independent variable to examine potential national differences in our findings. However, as shown later, national differences in credit and blame ratings were revealed through both ANOVAs and ANCOVAs in exploratory analyses.

### Credit attribution for beneficial outcomes

**FIGURE 2:**
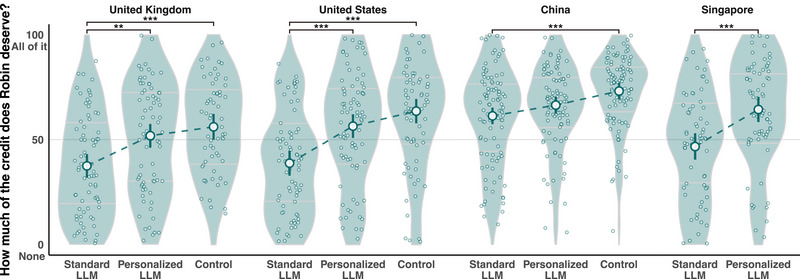
Credit attribution to Robin in three conditions in each country. *p < 0.05, **p < 0.01, ***p < 0.001.

When the content of Robin's blogpost was described as being full of useful information that could be helpful to many people, the content production method significantly influenced credit attributions among participants in the United Kingdom (*F*_(2, 214)_ = 11.02, *p* < 0.001, *η* ^2^_p_ = 0.09), United States (*F*_(2, 229)_ = 18.14, *p* < 0.001, *η* ^2^_p_ = 0.14), China (*F*_(2, 291)_ = 8.74, *p* < 0.001, *η* ^2^_p_ = 0.06), and Singapore (*F*_(1, 139)_ = 16.19, *p* < 0.001, *η* ^2^_p_ = 0.10). As shown in Figure 2, pairwise comparisons indicated that participants from the United Kingdom, United States, and Singapore attributed more credit to Robin when the protagonist used the personalized versus standard LLM for generating identical beneficial outcomes (all adjusted *p *< 0.05; see Table 1). However, Chinese participants did not attribute more credit to Robin for using the personalized LLM compared to the standard one (Δ*M* = 5.13, *t *= 1.80, *p* = 0.217, Cohen's *d* = 0.26). Thus, the data were consistent with **H1** in the United Kingdom, United States, and Singapore, but not in China. Furthermore, similar results were obtained after controlling for the above‐described individual difference measures through ANCOVAs in each nation (see Supporting Information tables). Thus, the national differences in **H1** were not considered attributable to participants’ individual differences in these questions.

**TABLE 1:** Summary statistics for each comparison.

Country	Outcome	Contrast	△*M*	*t*	*p*	Cohen's *d*
United Kingdom	Beneficial	Control—standard	18.51	4.38	<0.001	0.74
		Control—personalized	4.19	0.99	0.967	0.17
		Standard—personalized	−14.31	−3.57	0.001	−0.58
	Harmful	Control—standard	−11.39	−4.02	<0.001	−0.67
		Control—personalized	−13.21	−4.78	<0.001	−0.78
		Standard—personalized	−1.81	−0.68	1.000	−0.11
United States	Beneficial	Control—standard	24.72	5.86	<0.001	0.96
		Control—personalized	7.16	1.75	0.246	0.28
		Standard—personalized	−17.56	−4.23	<0.001	−0.68
	Harmful	Control—standard	−5.45	−2.04	0.128	−0.33
		Control—personalized	−2.72	−1.00	0.956	−0.16
		Standard—personalized	2.73	1.03	0.912	0.16
China	Beneficial	Control—standard	11.83	4.17	<0.001	0.59
		Control—personalized	6.70	2.35	0.058	0.34
		Standard—personalized	−5.13	−1.80	0.217	−0.26
	Harmful	Control—standard	3.10	1.44	0.453	0.20
		Control—personalized	−2.23	−1.03	0.915	−0.14
		Standard—personalized	−5.33	−2.45	0.045	−0.34
Singapore	Beneficial	Standard—personalized	−17.60	−4.02	<0.001	−0.68
	Harmful	Standard—personalized	−3.71	−1.10	0.275	−0.18

*Note*: The *p* values in multiple comparisons were adjusted by the Bonferroni method.

Although we did not preregister a prediction regarding the control condition (no LLM), our previous theorizing assumed that any LLM use might lower credit attributions for beneficial outcomes compared to control. However, results from all three nations with data in the control condition (i.e., excluding Singapore) showed that, although Robin was indeed attributed less credit when using the standard LLM versus the control condition (all *p* < 0.001), credit attribution for using the personalized LLM was not significantly below control (all *p* > 0.05; see Figure 2). In other words, Robin was attributed about as much credit for bringing about a beneficial outcome with the assistance of a personalized LLM as when the same benefit was produced “manually” (i.e., without the assistance of generative AI). These results remained after controlling for the three covariates in each nation.

### Blame attribution for harmful outcomes

**FIGURE 3:**
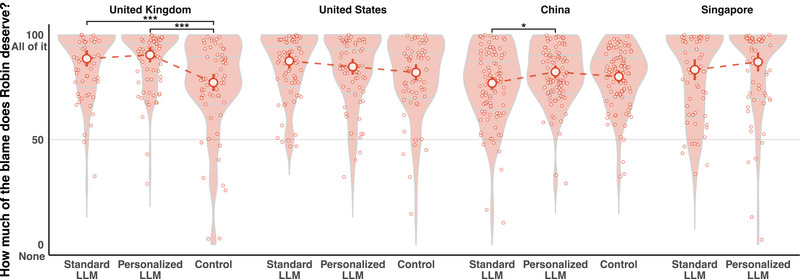
Blame attribution to Robin in three conditions in each country (apart from the control condition in Sinagpore). *p < 0.05, **p < 0.01, ***p < 0.001.

When the content of Robin's blogpost was described as being full of disinformation that could be harmful to many people, we found a significant influence of content production method (i.e., LLM type or control) on blame attributions in the United Kingdom (*F*_(2, 228)_ = 12.85, *p* < 0.001, *η* ^2^_p_ = 0.10) and China (*F*_(2, 306)_ = 3.03, *p* = 0.050, *η* ^2^_p_ = 0.02), but not in the United States (*F*_(2, 228)_ = 2.08, *p* = 0.128, *η* ^2^_p_ = 0.02) or Singapore (*F*_(1, 145)_ = 1.20, *p* = 0.275, *η* ^2^_p_ < 0.01). As shown in Figure 3, the difference in blame attributions between the personalized and standard LLM conditions was not significant in the United Kingdom, United States, and Singapore (all *p* > 0.200), except in China where Robin was deemed more blameworthy for using the personalized LLM (Δ*M* = 5.33, *t* = 2.45, *p* = 0.045, *d* = 0.34). These results remained consistent after controlling for the three covariates (see the Supporting Information tables), except that the difference in the Chinese sample became nonsignificant (*p* = 0.096). Thus, the data were largely consistent with **H2**, albeit with caution advised when interpreting data from Chinese participants.

As with the beneficial outcome conditions, we did not preregister a prediction regarding the control condition (no LLM). However, we found that LLM use (compared to no LLM) did not significantly influence blame attributions in two of the three countries for which we had final data in the control condition, namely, the United States and China (all *p* > 0.100; see Figure 3). By contrast, UK participants did attribute more blame to Robin for using either type of AI: both the standard LLM (Δ*M* = 11.39, *t *= 4.02, *p* < 0.001, *d* = 0.67) and the personalized LLM (Δ*M* = 13.21, *t* = 4.78, *p* < 0.001, *d* = 0.78) compared to the control condition. The significance of these comparisons remained robust even after controlling for the three covariates.

## DICUSSION AND CONCLUSION

As human–AI collaboration becomes more prevalent, it is crucial to understand the implications of LLM use on human responsibility for AI‐generated content. Here, we asked the general public in four countries how much credit or blame they attributed to a character named Robin, who brought about a positive or negative outcome using a standard LLM, personalized LLM, or no LLM (as a control). We hypothesized that more credit would be attributed to Robin for producing a beneficial outcome when using a personalized versus standard LLM (**H1),** whereas blame attributions for a given harmful outcome would be comparable regardless of LLM type (**H2**).

We found support for **H1** in three nations, with an exception in China (see Figure 2). In other words, the character's use of a “standard” LLM to produce a positive outcome resulted in lower credit ratings, not only in comparison to a personalized LLM, as specifically hypothesized, but also in comparison to the AI‐unassisted control. This is consistent with previous work documenting diminished praise for technologically enhanced human achievements, where this finding was attributed to a perceived lack of effort by the human user.^14^

When asked to justify their ratings of credit and blame using an open‐ended response box, some participants attributed their low ratings for a beneficial outcome generated by a standard LLM to a perceived lack of effort on Robin's part, whereas others attributed their low ratings to a perceived lack of creativity from Robin. Participants noted that “the product was given to [Robin] with minimum creativity” (US male, age 67), and that “every word or every sentence is not [Robin's] own emotions” (Chinese female, age 24). Other participants emphasized the “work” put in by the LLM: For example, “the LLM has all of the information and did all of the work, there was little thought or skill that originated from Robin” (US male, age 59).

Personalization, however, altered such perceptions. In the personalized LLM conditions, when the blogpost included helpful information, participants noted that the output reflected Robin's past writings and original ideas from the training data: “It's Robin's own work that the LLM [is] based on, not statistically derived from the masses” (Singaporean female, age 48). The output “was mainly composed of the author's own data, while the AI tool just played a role in assisting with summarization” (Chinese female, age 67). Participants further noted that the published piece “would not exist without Robin's initial thoughts and work” (UK female, age 38) and that without Robin's work, “the LLM was unlikely to create this useful blog” (Chinese female, age 20). As one participant put it, “If it's Robin's work that the AI was trained on, then they can take a lot of credit for the output” (UK male, age 32). That is, the output of Robin's personalized LLM was considered more original to Robin than the output generated by Robin using a standard LLM. We note, however, that these quotes are presented for illustrative purposes and do not purport to represent the statistical differences in participants’ reasoning across conditions.

Moreover, the credit attributed to Robin for bringing about a positive outcome by using a personalized LLM was statistically indistinguishable from that attributed to Robin in the control condition (no LLM). Thus, personalization may at least partially address concerns over what we and our colleagues have termed the AI “achievement gap”^5^, ^10^: “good, useful outcomes will be produced, but many of them will not be achievements for which human workers and professionals can claim credit.”^5^ In essence, by drawing on individual users’ previous creditworthy behavior to generate a new beneficial outcome—rather than drawing solely on others’ past work, as with standard LLMs—personalized LLMs may support the attribution of credit to users for novel, positive outcomes.

If so, it may also follow that rights and interests, including authorship and intellectual property rights, over generated output may be easier to claim when using personalized, rather than standard, generative AI, as the recognition of these rights and interests depends on the degree of skill and labor involved in generating an output.

There is another side to the coin, however. Although participants in three nations (United States, United Kingdom, and Singapore) attributed comparable blame to Robin using a standard LLM and a personalized LLM for an identical harmful output (thus supporting **H2**), Chinese participants were more likely to blame Robin for using the personalized LLM (although this effect was no longer statistically significant after controlling for covariates). Furthermore, UK participants unexpectedly attributed significantly more blame to Robin for using either type of LLM, compared to no LLM.

**H2** is consistent with the assumption^5^ that a human user needs only to be reckless or negligent in their use of a technology to be highly blameworthy for foreseeable harms. In all conditions in which the blogpost included potentially harmful disinformation, participants noted that Robin should have taken greater care to properly vet the blogpost before publishing it, rather than having “blind faith in technology” (Singaporean female, age 32). For instance, according to one participant, “If you publish it under your name, it's your responsibility. It doesn't matter how it was created” (US male, age 36).

Our work points toward future research avenues. First, current data do not explain the unexpectedly higher ratings of blame for personalized LLM use in the Chinese sample or for both types of LLM use (vs. control) in the UK sample. If such findings turn out to be robust and replicable, they might reflect an assumption, on the part of at least some participants, that any negative output from a personalized LLM could be due to the presence of blameworthy elements (e.g., errors, biases, or misinformation) in the user's own past work. Such an assumption would not be unreasonable, and it points to a real concern about personalization; that it could, in some cases, simply reinforce or even exacerbate biases or other problems that exist within an individual's body of work. Further research is needed to evaluate this potential explanation. Second, although we observed a clear pattern of heightened credit attributions for beneficial outcomes following from personalized versus standard LLM use and speculated that this could be due to assumptions about the user's previous creditworthy behavior, teasing apart different potential causes (e.g., creativity attribution vs. effort attribution, or the relative weights assigned to each) will require further empirical studies.

In summary, we found elevated credit attributions for personalized compared to standard LLM use, with LLM type making a smaller difference to blame judgments, albeit with subtle differences and exceptions across countries. Our results shed light on the complexity and nuance surrounding questions of credit and blame attribution for generative AI use. Further work is required to understand the nature and generalizability of our findings, which will be crucial for informed policymaking on generative AI use in relation to written work, art, music, and other creative endeavors.

## AUTHOR CONTRIBUTIONS

Brian D. Earp, Sebastian Porsdam Mann, Julian Savulescu, and Ivar Hannikainen conceived the idea. Brian D. Earp, Sebastian Porsdam Mann, Ivar Hannikainen, and Maryam Ali Khan designed the survey. Brian D. Earp, Maryam Ali Khan, and Yueying Chu carried out the survey. Ivar Hannikainen, Yueying Chu, Brian D. Earp, and Peng Liu analyzed the data. Sebastian Porsdam Mann, Brian D. Earp, Maryam Ali Khan, Ivar Hannikainen, and Peng Liu contributed to the writing. Julian Savulescu supervised the work. All authors contributed to the interpretation of the findings and multiple rounds of draft revision.

## CONFLICT OF INTEREST STATEMENT

Julian Savulescu is a bioethics committee consultant for Bayer and he is also a bioethics advisor to the Hevolution Foundation. Sebastian Porsdam Mann is a member of the ethics advisory board for Retroviral Therapeutics LLC and he is also a member of the advisory board for AminoChain Inc.

## Supporting information



Supporting Information

## Data Availability

Our data, materials, preregistration information, and code are publicly available (https://osf.io/jqte6/?view_only=a327a237393749d9b816c346d8965c95).
