# Supplementary material for: Credit and blame for AI–generated content: Effects of personalization in four countries
Source: Ann N Y Acad Sci. 2024 Nov 25;1542(1):51–7. doi: 10.1111/nyas.15258 (PMC11668494; doi:10.1111/nyas.15258)
Supplement: Supplementary file 1 — Supporting Information [file NYAS-1542-51-s001.docx]

# *Supporting Information for*

**Credit and Blame for AI-Generated Content: Effects of Personalization in Four Countries**

Brian D. Earp, Sebastian Porsdam Mann, Peng Liu, Ivar Hannikainen, Maryam Ali Khan, Yueying Chu, and Julian Savulescu

## Supplementary Note S1. Open Science and Power Analysis

Using the *pwr* package in *R*, we established a target sample size of 354 in order to reliably detect a small effect (Cohen’s *f =* 0.20) with 90% statistical power and an alpha level of .05 and 3 numerator degrees of freedom (for a 2 x 2 design; excluding control conditions). Anticipating exclusions, and with the addition of two control conditions, we aimed to recruit slightly more than 600 participants in each country.

## Supplementary Note S2. Participant Description/Details

Our samples consisted of 640 UK participants, 667 Chinese participants, 682 Singaporean participants, and 644 US participants. Final samples determined after data exclusions can be found in Supplementary Table 1.

## Supplementary Table S1. Participant characteristics by country

| **Country** | **Total Participants** | | | | **Age** | |
| --- | --- | --- | --- | --- | --- | --- |
|  |  | **Men** | **Women** | **“Non-binary/Other” or “Prefer not to say” or no response** | ***Age, Mean*** | ***SD*** |
| UK | 448 | 225 | 220 | 3 | 46.5 | 15.4 |
| US | 463 | 216 | 236 | 11 | 46.7 | 16.1 |
| China | 603 | 299 | 304 | - | 44.0 | 13.9 |
| Singapore | 288 | 122 | 166 | - | 46.5 | 13.4 |

*Note:* data from the two control conditions in the Singapore sample were excluded due to a programming error, as mentioned in the main text.

## Supplementary Note 3. Vignettes

### LLM Explanation

Prior to reading the vignettes, all participants in the LLM conditions were shown an explanation of what an LLM is and how it works. This explanation varied slightly depending on which LLM condition (standard/personalized) participants were assigned to (see Figure 1 for the standard LLM explanation). In the control conditions, participants were only given the explanatory LLM information *after* making their credit and blame ratings in relation to the blogpost written without the aid of any LLM. In the Singapore study, however, this order-change was not implemented due to a programming error, meaning that Singapore participants in the control conditions were erroneously shown information about LLMs prior to making their credit and blame ratings. Therefore, as noted in the main text, the data from the two Singapore control conditions were excluded prior to data analysis.

In the personalized LLM condition, after the general explanation of what an LLM is and how it works, the text continued as follows:

*However, it is also now possible to "fine-tune" an LLM to a specific body of work – for example, the collected writings of a single author. Such a "personalized" LLM has the same basic training as a standard LLM, but with an added layer of statistics that captures the more specific patterns of information embedded in the writings of a single author.*

### Vignettes

We designed four vignettes based on a 3 (two LLMs + control) x 2 (outcome type) design, which were then presented to participants. These entailed a fictitious character, Robin, who either used an LLM to write a blog post, or, in the control condition, wrote the post without help from an LLM, using information from the internet. All participants were randomly assigned to one of the three LLM conditions (personalized, standard, or control), and to one of two outcome conditions reflecting the effects of the blog post, described as either beneficial or harmful.

The text for the vignettes differed based on the description of the LLM, or control. The text used for the outcomes was the same across all conditions (see Supplementary Table 2).

##

## Supplementary Table S2. Vignette text by condition and outcome

| **Two LLMs + Control** | **Main Text** | **Beneficial Outcome** | **Harmful Outcome** |
| --- | --- | --- | --- |
| **Standard LLM** | *Now imagine the following scenario:*    *Robin has generated a blog post using a standard LLM that is trained on a huge corpus of text from the internet. Robin entered some short prompts into the program and the LLM was able to produce the blog post.* | *After quickly skimming it over, Robin publishes the post online.* ***As it happens, the post offers really useful, positive insights that could benefit lots of people.***    ***Suppose the blog post actually goes on to cause a great deal of benefit. How much of the credit does Robin deserve to take for the beneficial outcome of the blog post?’*** | *After quickly skimming it over, Robin publishes the post online.* ***As it happens, the post includes lots of disinformation that could be very harmful to lots of people.***  ***Suppose the blog post actually goes on to cause a great deal of harm. How much of the blame does Robin deserve to take for the harmful outcome of the blog post?*** |
| **Personalized LLM** | *Now imagine the following scenario:*    *Robin has generated a blog post using a personalized LLM that is trained on Robin's own prior research and writing. Robin entered some short prompts into the program and the LLM was able to produce the blog post.* |  |  |
| **Control** | *Imagine the following scenario:*    *Robin has written a blog post using information from the internet. Robin entered some short prompts into the search engine and was able to produce the blog post based on the results that came up.* |  |  |

## Supplementary Note S4. Measures and Attention Check

### Comprehension checks

The four LLM vignettes were preceded by a comprehension check which entailed selecting the correct explanation of how an LLM works; in the personalized LLM conditions, a definition of both standard and personalized LLMs was provided. In the control conditions, an explanation of a standard LLM was provided after the vignette.

### Attribution of credit and blame

After reading the vignette, all participants rated how much blame (or credit) Robin deserved on a scale of 0 (none at all) to 100 (all of it) for the harmful (or beneficial) outcome of the blog post.

### Attention check

To ensure that participants attentively read the vignette and provided blame (or credit) ratings, the attention check asked them to indicate what the previous question had asked them to do.

### AI-related questions

**LLM experience.** Participants were asked to indicate their experience with using LLMs such as ChatGPT on a scale from 0 (no experience) to 100 (a lot of experience).

**AI replacement concern.** Participants were asked to rate how worried they were about AI replacing humans in creative fields on a scale from 0 (not worried) to 100 (very worried).

**Technological propensity (i.e., attitudes about being informed about technological advancement).** Participants were asked to rate how much they liked or disliked being informed about technological advancements such as chatbots and AI, on a scale from 0 (strongly dislike) to 100 (strongly like).

## Supplementary Note S5. Chinese Translation

### Chinese Survey Translation

For the Chinese survey, we conducted an LLM-assisted “back-translation” in two steps. In Step 1, two Chinese researchers translated the original English into Chinese. In Step 2 (translated Chinese to translated English), they utilized GPT 3.5 from OpenAI to generate five different versions of the translated English surveys based on the translated Chinese survey. To assess the lexical similarity, we employed the BiLingual Evaluation Understudy metric (BLEU; Papineni et al., 2002), which automatically evaluates machine-translated text. The average BLEU-1 score of the five versions equaled or slightly exceeded 0.50 (see Appendix 3). According to its associated rule of thumb (Papineni et al., 2002), this indicates that the translated English versions exhibited acceptable translation quality and further suggests the acceptability of the Chinese version. It is essential to note that lexical similarity measured by BLEU does not equate to semantic similarity. Nonetheless, based on our experience, we determined that the Chinese survey demonstrates acceptable semantic similarity.

## Supplementary Table S3. BLEU score (Standard LLM x Beneficial Outcome)

| **Different versions of ChatGPT translation** | **BLEU-1** | **BLEU-2** | **BLEU-3** | **BLEU-4** |
| --- | --- | --- | --- | --- |
| V1 | 0.60 | 0.43 | 0.32 | 0.22 |
| V2 | 0.50 | 0.34 | 0.26 | 0.18 |
| V3 | 0.58 | 0.43 | 0.33 | 0.24 |
| V4 | 0.54 | 0.36 | 0.25 | 0.16 |
| V5 | 0.56 | 0.40 | 0.31 | 0.22 |

## Supplementary Table S4. BLEU score (Standard LLM x Harmful Outcome)

| **Different versions of ChatGPT translation** | **BLEU-1** | **BLEU-2** | **BLEU-3** | **BLEU-4** |
| --- | --- | --- | --- | --- |
| V1 | 0.56 | 0.40 | 0.29 | 0.20 |
| V2 | 0.52 | 0.37 | 0.27 | 0.19 |
| V3 | 0.57 | 0.41 | 0.30 | 0.21 |
| V4 | 0.55 | 0.40 | 0.30 | 0.21 |
| V5 | 0.59 | 0.40 | 0.28 | 0.18 |

## Supplementary Table S5. BLEU score (Personalized LLM x Beneficial Outcome)

| **Different versions of ChatGPT translation** | **BLEU-1** | **BLEU-2** | **BLEU-3** | **BLEU-4** |
| --- | --- | --- | --- | --- |
| V1 | 0.53 | 0.38 | 0.28 | 0.20 |
| V2 | 0.52 | 0.37 | 0.28 | 0.19 |
| V3 | 0.55 | 0.39 | 0.29 | 0.20 |
| V4 | 0.53 | 0.36 | 0.25 | 0.16 |
| V5 | 0.56 | 0.38 | 0.27 | 0.19 |

##

## Supplementary Table S6. Pairwise comparisons of credit/blame attribution between LLM conditions and control, without covariates

| Country | Outcome | Contrast | △*M* | *T* | *p* | Cohen’s *d* |
| --- | --- | --- | --- | --- | --- | --- |
| United  Kingdom | Beneficial | Control - Standard | 18.51 | 4.38 | < .001 | 0.74 |
|  |  | Control - Personalized | 4.19 | 0.99 | .967 | 0.17 |
|  |  | Standard - Personalized | −14.31 | −3.57 | .001 | −0.58 |
|  | Harmful | Control - Standard | −11.39 | −4.02 | < .001 | −0.67 |
|  |  | Control - Personalized | −13.21 | −4.78 | < .001 | −0.78 |
|  |  | Standard - Personalized | −1.81 | −0.68 | 1.000 | −0.11 |
| United  States | Beneficial | Control - Standard | 24.72 | 5.86 | < .001 | 0.96 |
|  |  | Control - Personalized | 7.16 | 1.75 | .246 | 0.28 |
|  |  | Standard - Personalized | −17.56 | −4.23 | < .001 | −0.68 |
|  | Harmful | Control - Standard | −5.45 | −2.04 | .128 | −0.33 |
|  |  | Control - Personalized | −2.72 | −1.00 | .956 | −0.16 |
|  |  | Standard - Personalized | 2.73 | 1.03 | .912 | 0.16 |
| China | Beneficial | Control - Standard | 11.83 | 4.17 | < .001 | 0.59 |
|  |  | Control - Personalized | 6.70 | 2.35 | .058 | 0.34 |
|  |  | Standard - Personalized | −5.13 | −1.80 | .217 | −0.26 |
|  | Harmful | Control - Standard | 3.10 | 1.44 | .453 | 0.20 |
|  |  | Control - Personalized | −2.23 | −1.03 | .915 | −0.14 |
|  |  | Standard - Personalized | −5.33 | −2.45 | .045 | −0.34 |
| Singapore | Beneficial | Standard - Personalized | −17.60 | −4.02 | < .001 | −0.68 |
|  | Harmful | Standard - Personalized | −3.71 | −1.10 | .275 | −0.18 |

*Note*. The *p* values in multiple comparisons were adjusted by the Bonferroni method.

## Supplementary Table S7. Pairwise comparisons of credit/blame attribution between LLM conditions and control, with covariates (LLM experience, technological propensity and AI replacement concern)

| Country | Outcome | Contrast | △*M* | *T* | *p* | Cohen’s *d* |
| --- | --- | --- | --- | --- | --- | --- |
| United  Kingdom | Beneficial | Control - Standard | 18.78 | 4.40 | < .001 | 0.77 |
|  |  | Control - Personalized | 5.42 | 1.26 | .627 | 0.22 |
|  |  | Standard - Personalized | −13.35 | −3.36 | .003 | −0.55 |
|  | Harmful | Control - Standard | −12.20 | −4.21 | < .001 | −0.72 |
|  |  | Control - Personalized | −13.50 | −4.86 | < .001 | −0.79 |
|  |  | Standard - Personalized | −1.31 | −0.48 | 1.000 | −0.08 |
| United  States | Beneficial | Control - Standard | 24.28 | 5.93 | < .001 | 0.97 |
|  |  | Control - Personalized | 5.78 | 1.45 | .449 | 0.23 |
|  |  | Standard - Personalized | −18.50 | −4.56 | < .001 | −0.74 |
|  | Harmful | Control - Standard | −5.12 | −1.91 | .173 | −0.31 |
|  |  | Control - Personalized | −3.44 | −1.27 | .619 | −0.21 |
|  |  | Standard - Personalized | 1.68 | 0.64 | 1.000 | 0.10 |
| China | Beneficial | Control - Standard | 11.60 | 4.29 | < .001 | 0.62 |
|  |  | Control - Personalized | 5.76 | 2.08 | .115 | 0.31 |
|  |  | Standard - Personalized | −5.84 | −2.15 | .097 | −0.31 |
|  | Harmful | Control - Standard | 3.01 | 1.41 | .478 | 0.20 |
|  |  | Control - Personalized | −1.51 | −0.70 | 1.000 | −0.10 |
|  |  | Standard - Personalized | −4.52 | −2.08 | .115 | −0.29 |
| Singapore | Beneficial | Standard - Personalized | −18.50 | −4.33 | < .001 | −0.74 |
|  | Harmful | Standard - Personalized | −4.19 | −1.29 | .198 | −0.22 |

*Note*. The *p* values in multiple comparisons were adjusted by the Bonferroni method.

## Supplementary Note S6. Description of statistical methods.

Statistical analyses were conducted in R, version 4.3.1, using *car*, *emmeans*, and *effectsize* packages. We conducted analyses of variance (ANOVA) among three levels of the LLM condition in each country at each level of the outcome. Then, given that participants’ individual differences (e.g., LLM experience, AI replacement concern, and technological propensity) might influence their credit and blame ratings, we conducted analyses of covariance (ANCOVA), treating these individual differences as covariates. Bonferroni correction was used for multiple comparisons. A complete analysis script is available on the *Open Science Framework* at: <https://osf.io/jqte6/>.

## Supplementary Note 7. Results of the 3×2 ANOVA mentioned in the pre-registration form.

As noted in the main text, we pre-registered a 3 (two LLM conditions and one control condition) × 2 (outcome: beneficial and harmful) ANOVA in each country (see the results in Supplementary Table 8). After collecting the data, we realized that a direct comparison of ratings from the two outcome conditions might not be reasonable, since, strictly speaking, the ratings were collected on different scales (i.e., one asking about how much *blame* Robin deserved; the other asking about how much *credit* Robin deserved). In the first submitted draft of the manuscript we therefore decided against including results from the planned ANOVA, choosing instead to conduct separate ANOVAs on each outcome variable (one for the harmful outcome conditions, one for the beneficial outcomes conditions). Nevertheless, in response to a request from a peer reviewer that we include the results from the originally planned analysis (noting that there is disagreement in the field about whether credit and blame judgments can be directly compared, despite being measured on different scales), and notwithstanding our reservations, we now briefly mention the results in the main text and give a more complete description here.

Given that the interaction between the two independent variables was significant in each country, their simple effects should be examined. Our main manuscript reports the influences of LLM condition. In this supplementary file, we only report the simple effects of outcome (i.e., the difference between credit and blame attributions in each LLM condition). In all LLM conditions and surveyed countries, more blame was assigned to Robin for the harmful outcome than credit assigned to Robin for the beneficial outcome.

## Supplementary Table S8. Results of two-way ANOVA.

|  | UK | | | US | | | China | | | Singapore | | |
| --- | --- | --- | --- | --- | --- | --- | --- | --- | --- | --- | --- | --- |
|  | *F* | *p* | η²_p_ | *F* | *p* | η²_p_ | *F* | *p* | η²_p_ | *F* | *p* | η²_p_ |
| LLM condition | 5.76 | .003 | .025 | 8.27 | < .001 | .035 | 9.38 | < .001 | .030 | 14.97 | < .001 | .050 |
| Outcome | 335.71 | < .001 | .432 | 246.81 | < .001 | .351 | 77.51 | < .001 | .115 | 115.51 | < .001 | .289 |
| LLM condition  × Outcome | 17.64 | < .001 | .074 | 19.16 | < .001 | .077 | 4.14 | .016 | .014 | 6.36 | .012 | .022 |

*UK.* Outcome (credit vs. blame attributions) had significant effects in each LLM condition (control: *F*_(1, 442)_ = 32.45, *p* < .001, η²_p_ = .068; standard LLM: *F*_(1, 442)_ = 223.02, *p* < .001, η²_p_ = .335; personalized LLM: *F*_(1, 442)_ = 134.14, *p* < .001, η²_p_ = .233). In the UK sample, more blame was assigned to Robin for the harmful outcome than credit assigned to Robin for the beneficial outcome (control: Δ*M* = 21.11, *t*_(442)_ = 5.70, *p* < .001, *d* = 1.00; standard LLM: Δ*M* = 51.01, *t*_(442)_ = 14.93, *p* < .001, *d* = 2.41; personalized LLM: Δ*M* = 38.51, *t*_(442)_ = 11.58, *p* < .001, *d* = 1.82).

*US.* Outcome (credit vs. blame attributions) had significant effects on LLM conditions and control condition (control: *F*_(1, 457)_ = 26.90, *p* < .001, η²_p_ = .056; standard LLM: *F*_(1, 457)_ = 193.15, *p* < .001, η²_p_ = .297; personalized LLM: *F*_(1, 457)_ = 66.88, *p* < .001, η²_p_ = .128). In the US sample, more blame was assigned to Robin for the harmful outcome than credit assigned to Robin for the beneficial outcome (control: Δ*M* = 18.40, *t*_(457)_ = 5.19, *p* < .001, *d* = 0.85; standard LLM: Δ*M* = 48.58, *t*_(457)_ = 13.90, *p* < .001, *d* = 2.24; personalized LLM: Δ*M* = 28.28, *t*_(457)_ = 8.18, *p* < .001, *d* = 1.30).

*China.* Outcome (credit vs. blame attributions) had significant effects on LLM conditions and control condition (control: *F*_(1, 597)_ = 7.58, *p* = .006, η²_p_ = .013; standard LLM: *F*_(1, 597)_ = 39.03, *p* < .001, η²_p_ = .061; personalized LLM: *F*_(1, 597)_ = 38.85, *p* < .001, η²_p_ = .061). In the Chinese sample, more blame was assigned to Robin for the harmful outcome than credit assigned to Robin for the beneficial outcome (control: Δ*M* = 6.89, *t*_(597)_ = 2.75, *p* = .006, *d* = 0.39; standard LLM: Δ*M* = 15.62, *t*_(597)_ = 6.25, *p* < .001, *d* = 0.88; personalized LLM: Δ*M* = 15.82, *t*_(597)_ = 6.23, *p* < .001, *d* = 0.89).

*Singapore.* Outcome (credit vs. blame attributions) had significant effects on LLM conditions (standard LLM: *F*_(1, 284)_ = 82.37, *p* < .001, η²_p_ = .225; personalized LLM: *F*_(1, 284)_ = 36.33, *p* < .001, η²_p_ = .113). In the Singapore sample, more blame was assigned to Robin for the harmful outcome than credit assigned to Robin for the beneficial outcome (standard LLM: Δ*M* = 36.49, *t*_(284)_ = 9.08, *p* < .001, *d* = 1.57; personalized LLM: Δ*M* = 22.62, *t*_(284)_ = 6.03, *p* < .001, *d* = 0.97).

**Supplementary Note S8.** The need for further study of between-country differences.

In the main text, we report subtle differences between some of the countries in terms of their credit or blame ratings. However, due to space limitations and adherence to our pre-registration (we did not pre-register any hypotheses or analyses about between-country differences), we do not expand on these differences in the manuscript. We will offer some speculative thoughts here in the Supplemental File, but please note that further research will be required to investigate this matter directly.

One the one hand, it seems to us that between-country differences in credit or blame ratings may be linked to the differences in participants’ attitudes toward LLMs and AI technology. For example, it may be that people from developing countries are more positive toward AI technology and thus may be more willing to assign credit to their users. As shown in Supplementary Table 9 below, although there are inconsistent correlations between the three individual difference measures (LLM experience, AI replacement concern, and technological propensity) and credit attributions across the surveyed samples, it can be seen that credit attribution is positively correlated with technological propensity and LLM experience and negatively correlated with AI replacement concern. In our case, that the Chinese sample was more willing to attribute credit to Robin in the two LLM conditions may be linked to their more positive attitude toward new technology; for instance, as compared to the US sample, the Chinese sample in two LLM conditions showed less AI replacement concern (Δ*M* = −10.59, *t*_(710)_ = −5.20, *p* < .001, *d* = −0.39) and reported more experience with LLMs (Δ*M* = 11.66, *t*_(709)_ = 6.00, *p* < .001, *d* = 0.45).

**Supplementary Table S9**. Correlations between three individual difference measures (LLM experience, AI replacement concern, and technological propensity) and blame/credit attributions across the four nations.

| United Kingdom | | | | | United States | | | | |
| --- | --- | --- | --- | --- | --- | --- | --- | --- | --- |
|  | TP | RC | LLM Exp | Blame |  | TP | RC | LLM Exp | Blame |
| TP |  | −.17^*^ | .38^***^ | .10 | TP |  | −.10 | .23^**^ | .10 |
| RC | −.03 |  | −.09 | .10 | RC | −.31^***^ |  | −.23^**^ | .00 |
| LLM Exp | .27^***^ | −.03 |  | −.19* | LLM Exp | .31^***^ | −.17^*^ |  | −.17^*^ |
| Credit | .11 | −.19^*^ | .04 |  | Credit | .25^**^ | −.12 | .16^*^ |  |
| China | | | | | Singapore | | | | |
|  | TP | RC | LLM Exp | Blame |  | TP | RC | LLM Exp | Blame |
| TP |  | −.16^*^ | .51^***^ | .20^**^ | TP |  | .11 | .24^**^ | .37^***^ |
| RC | −.25^***^ |  | −.18^**^ | .12 | RC | .19^*^ |  | −.08 | .06 |
| LLM Exp | .51^***^ | −.16^*^ |  | .10 | LLM Exp | .40^***^ | .04 |  | .09 |
| Credit | .34^***^ | −.20^**^ | .15^*^ |  | Credit | .16 | −.09 | .19^*^ |  |

*Note*: TP = technological propensity, RC = AI replacement concern, LLM Exp = LLM experience, Credit = Credit attribution, Blame = Blame attribution. **p* < 0.05, ***p* < 0.01, ****p* < 0.001.

On the other hand, we cannot rule out that language translation may have influenced some of the observed differences across the four nations. Although the US, UK, and Singapore experiments were conducted in English, the Chinese experiment used English-to-Chinese translated materials as explained above. As seen Figure 2 in the main text, the Chinese sample was more willing to attribute credit to Robin not only in the two LLM conditions but also in the control condition. Possibly, the words for “credit” and “blame” (“赞扬” and “责备” in Chinese) may have different connotations in different languages, leading participants to respond differently to identical items in different languages (e.g., David et al., in press). Further research will be required to investigate such possibilities.

## Reference

David, L., Vassena, E., & Bijleveld, E. (in press). The unpleasantness of thinking: A meta-analytic review of the association between mental effort and negative affect. *Psychological Bulletin*. <https://doi.org/10.1037/bul0000443>.

Papineni, K., Roukos, S., Ward, T., & Zhu, W.-J. (2002). BLEU: A method for automatic evaluation of machine translation. In *Proceedings of the 40th Annual Meeting on Association for Computational Linguistics*, 2002, Philadelphia, Pennsylvania.
